# Spanlastics as a Potential Approach for Enhancing the Nose-To-Brain Delivery of Piperine: In Vitro Prospect and In Vivo Therapeutic Efficacy for the Management of Epilepsy

**DOI:** 10.3390/pharmaceutics15020641

**Published:** 2023-02-14

**Authors:** Isha Gupta, Syeda Nashvia Adin, Md Abdur Rashid, Yahya Alhamhoom, Mohd. Aqil, Mohd. Mujeeb

**Affiliations:** 1 Phytomedicine Laboratory, Department of Pharmacognosy & Phytochemistry, School of Pharmaceutical Education & Research, Jamia Hamdard, New Delhi 110062, India; 2 Department of Pharmaceutics, College of Pharmacy, King Khalid University, Abha 62223, Saudi Arabia; 3 Department of Pharmaceutics, School of Pharmaceutical Education & Research, Jamia Hamdard, New Delhi 110062, India

**Keywords:** piperine, spanlastics, epilepsy, Box–Behnken design, blood–brain distribution

## Abstract

The present study delineates the preparation of piperine-loaded spanlastics (PIP-SPL) to improve piperine (PIP) solubility, bioavailability, and permeation through nasal mucosa for intranasal delivery. PIP-SPL was formulated using the thin-film hydration method and optimization was performed using Box–Behnken design (BBD). PIP-SPL optimized formulation (PIP-SPLopt) was characterized for polydispersity index (PDI), vesicle size, entrapment efficiency, zeta potential, and in vitro PIP release. For further evaluation, blood–brain distribution study, transmission electron microscopy (TEM), nasal permeation study, and confocal scanning laser microscopy (CLSM) were performed withal. The PIP-SPLopt presented spherical and sealed shape vesicles with a small vesicle size of 152.4 nm, entrapment efficiency of 72.93%, PDI of 0.1118, and in vitro release of 82.32%. The CLSM study unveiled that the developed formulation has greater permeation of PIP across the nasal mucosa in comparison with the PIP suspension. The blood–brain distribution study demonstrated higher C_max_ and AUC_0–24h_ of PIP-SPL via the intranasal route in comparison to PIP-SPL via oral administration. The in vivo study revealed that the PIP-SPL has good antiepileptic potential in comparison with the standard diazepam, which was evinced by seizure activity, neuromuscular coordination by rotarod test, biochemical estimation of oxidative stress markers, and histopathological studies. Furthermore, nasal toxicity study confirm that the developed PIP-SPL formulation is safer for intranasal application. The current investigation corroborated that the prepared spanlastic vesicle formulation is a treasured carrier for the PIP intranasal delivery for the management of epilepsy.

## 1. Introduction

Epilepsy is a chronic neurological disorder characterized by recurrent seizures, ascribable to the freakish electrical release of neurons, majorly in the hippocampus and neocortex in the brain [1]. It is a typical brain disorder afflicting 65 million people worldwide and precipitates other neuropsychiatric disorders such as depression and anxiety [2,3].

The major drugs used in epilepsy include benzodiazepines, valproic acid, gabapentin, lamotrigine, and pregabalin. Howbeit, their use has been restricted, owing to their untoward effects such as short-term memory loss, hyperactivity, visual problems, and drowsiness [4,5]. Ergo, herbal phytoconstituents have gained increased attention attributable to their natural abundance, low systemic toxicity, and therapeutic activities making them appropriate to be used in pharmaceutical preparations and food supplements [6,7,8]. Piperine (PIP) is an alkaloid that is chiefly present in plants: *Piper nigrum*, *Piper longum*, and *Piper officinarum* [9]. PIP has been copiously explored in diverse therapeutic fields due to its multifaceted biological profile that includes antihypertensive, antioxidant, antipyretic, antitumor, anti-asthmatics, analgesic, anti-inflammatory, antidiarrheal, anxiolytic, hepato-protective, antibacterial, antispasmodic, immunomodulatory, antifungal, neuroprotective, antimutagenic, and antimetastatic activity [10,11,12,13,14,15,16]. PIP manifests promising antiepileptic potential by dint of its antioxidant and neuroprotective potential [17]. Kaur et al. adumbrated that PIP acts by glycine and GABA signaling pathways and ameliorates oxidative stress markers [18].

Despite of its diverse therapeutic effects, its poor water solubility (0.04 mg/mL), poor permeability, low bioavailability and extensive first-pass metabolism delayed its transport across the blood–brain barrier and hence limits its use in emergency epileptic seizures [19]. Thereby, alternative routes are requisite to ameliorate seizure risk wherein oral administration is not probable. Owing to the specialized anatomical attributes of the nasal cavity, the intranasal route has been copiously explored to deliver the drugs directly to the brain through the trigeminal pathway and olfactory neuron, which will circumvent the blood–brain barrier (BBB), hepatic metabolism, and blood–cerebrospinal fluid barrier [20]. Nano-liposomal formulations have promising therapeutic effects, which deliver drugs promptly to the brain via nasal mucosa [21].

Several attempts have been made by researchers to enhance herbal drug permeability by delivering the drugs through conventional colloidal carriers like microemulsions, liposomes, solid-lipid nanoparticles or niosomes [22,23]. Howbeit, rigid structure, lack of flexibility and deformability during the delivery of drugs by these carriers via biological membrane limits their use. Therefore, recent studies have been investigated to augment the conventional carrier elasticity to increase the drug permeability via different biological membranes [24].

The spanlastic, a novel elastic nanovesicle carrier is a flexible system comprising an edge activator and a nonionic surfactant. Medication that is either hydrophobic or hydrophilic can be delivered through spanlastics, which are encapsulated in a compartment made by outer lipid and interior hydrophilic layers. Spanlastics are biodegradable, nonimmunogenic, and nontoxic vesicular carriers. Many studies have shown that spanlastics can augment drug bioavailability, therapeutic efficacy, patient compliance, and minimize adverse effects. Additionally, owing to their elasticity, these vesicular vehicles outperform liposomes in terms of chemical stability, and they have additional advantages over niosomal colloidal delivery systems. Furthermore, edge activator residence in the vesicle lipid membrane augments the nano-sized vesicles permeability by acting as a disrupting element and increasing the deformability athwart the biological membranes, all of which are recognized benefits of spanlastic vesicles’ elasticity [25].

Several studies have demonstrated that spanlastics can deliver specific drugs to the brain through nose-to-brain delivery. For example, Saleh et al. observed the “increased drug penetration across the nasal membrane in zolmitriptan-loaded spanlastic formulations, confirming the promising impact of intra-nasal dosing for brain delivery” [26]. Furthermore, Rehab et al. observed that “the formation of granisetron hydrochloride spanlastic gel appears to promote the brain bioavailability of the carried drug, providing a higher level of treatment in the brain” [27]. Yassin et al. also demonstrated that spanlastics can augment the intranasal delivery of carbamazepine to the CNS (brain) [28].

As a result, this study aims to combine the benefits of spanlastics delivery, nanotechnology’s ability and the leverage provided by the nose-to-brain delivery to augment the piperine nose-to-brain delivery for management of epilepsy.

Herein, this study is aimed at developing piperine-loaded modified spanlastics (PIP-SPL) by thin-film hydration method using phospholipon 90G, span 60, and sodium cholate. For the optimization of the developed formulation, a Box–Behnken design (BBD) was employed by taking phospholipon 90G, span 60, and sodium cholate as independent variables and their impact were assessed on dependent variables viz. polydispersity index (PDI), vesicle size, and entrapment efficiency (EE). Furthermore, the optimized formulation (PIP-SPLopt) was characterized for confocal scanning laser microscopy (CLSM), nasal permeation study, in vitro release kinetics, nasal toxicity, in vivo pharmacokinetic, and pharmacodynamics studies.

## 2. Materials and Methods

Piperine standard was acquired from Sigma Aldrich (Bangalore, India), Piperine API was procured from Yucca enterprises (Mumbai, India). Phospholipon 90G was procured from Lipoid (Ludwiashafen, Germany), sodium cholate was procured from DC Fine Chemicals (Mumbai, India), polyethylene glycol-400, methanol, chloroform span 60, and cholesterol were acquired from SD fine chemicals (Mumbai, India). Oxidative stress markers (superoxide dismutase, catalase, glutathione, malondialdehyde) kits were acquired from Abcam, Cambridge (UK). Pentylenetetrazole was procured from Sigma Aldrich (Bangalore, India). All HPLC solvents were acquired from SD Fine chemicals (Mumbai, India).

### 2.1. Formulation of Piperine Loaded Spanlastics (PIP-SPL)

The piperine loaded modified spanlastics were prepared using thin film hydration technique. In a round bottomed flask (RBF), 60 mg of phospholipon 90G (lipid), piperine (drug- 10 mg) and 15 mg of span 60 were dissolved in 10 mL of methanol: chloroform (1:3, *v*/*v*). The organic blend was evaporated under vacuum to attain a thin uniform layer of lipid deposited around the walls of RBF using rotary evaporator. The RBF was placed in desiccator for 24 h. The dried film was hydrated using solution of sodium cholate in nasal saline buffer (pH 6.5) for 1 h and stowed in refrigerator to acquire adequate swelling. The attained dispersion was probe sonicated for 4 min using titanium probe ultra sonicator (UP 100 H, Hielscher Ultrasonics GmbH, Berlin, Germany) and further characterized for PDI, entrapment efficiency, zeta potential, and vesicle size [20].

### 2.2. Optimization of Spanlastics Using Box–Behnken Design (BBD)

Preliminary screening trials were done to ascertain the potential parameters that impact the beneficial properties of spanlastics for intranasal delivery. After the determination of desirable parameters, a three-factor BBD was employed using Design Expert version 13 software (State-ease, Minneapolis, MN, USA). BBD was employed to ascertain the impact of lipid concentration, sodium cholate concentration, and span 60 concentration on response variables—PDI, entrapment efficiency, and vesicle size. The design comprised of 17 experimental runs (Table 2). Quadratic response surface model (generated by the Box–Behnken design) was as follows:

Z = k_0_ + k_1_ × 1 + k_2_X_2_ + k_3_X_3_ + k_12_X_1_X_2_ + k_13_X_1_X_3_ + k_23_X_2_X_3_ + k_11_X_12_ + k_22_X_22_ + k_33_X_32_

wherein Z—Predicted response;

Xi—the independent variables;

ki, ki_2_, ki_3_—quadratic, linear and interactive coefficients

The selected independent variables were phospholipon 90G concentration (X1), sodium cholate concentration (X3), and span 60 concentration (X2), and the dependent variables were PDI (Y1), vesicle size (Y2), and EE (Y3) (Table 1).

### 2.3. Characterization of PIP-SPLopt

#### 2.3.1. Vesicle Size, Zeta Potential, and Polydispersity Index (PDI)

The vesicle size, PDI and zeta potential of developed PIP-SPL were estimated using zeta sizer (Malvern instruments, Worcestershire, UK) after diluting the sample with Mill-Q water (50 times) at 25 ± 1 °C in triplicate at a scattering angle of 90 °C [20].

#### 2.3.2. Entrapment Efficiency

The ultracentrifugation method was employed to assess the entrapment efficiency of PIP-SPL [20]. The samples were stowed overnight at 4 °C and then subjected to centrifugation for 1 h at 4 °C using centrifuge at 20,000 rpm (REMI, cooling centrifuge, Mumbai, India). The filtrate, comprising free piperine, was separated and diluted with the appropriate medium and further the piperine content was assessed using HPLC. The entrapment efficiency was determined using the formula:

$$\%\;\mathrm{EE}=\frac{\mathrm{Total}\;\mathrm{piperine}-\;\mathrm{Piperine}\;\mathrm{in}\;\mathrm{supernatant}}{\mathrm{Total}\;\mathrm{piperine}}\times 100$$

The piperine content was assessed by HPLC analysis with mobile phase water: methanol (30:70) at a 1 mL/min flow rate using C18 column. UV detection wavelength was fixed at 343 nm [16].

### 2.4. Spanlastics Morphology

The morphology analysis of the PIP-SPLopt was carried out using transmission electron microscopy (TEM—Tecnai, CM 200, Philips scientific, Standford, CA, USA). Before analysis, on a copper grid, a drop of diluted sample was applied and then analyzed under transmission electron microscope after negatively staining with phosphotungstic acid (2%) [20].

### 2.5. In Vitro PIP Release Study

Drug release dialysis membrane technique was adopted to evaluate the in vitro release of PIP suspension (control) and PIP-SPLopt. Both formulations were thronged in the 12,000–14,000 Da preactivated dialysis membrane (Hi Media, Mumbai, Maharashtra, India), which was affixed with the shafts placed in a release medium of phosphate buffer saline (pH 6.5) contained in a beaker (500 mL), regulated at a temperature of 37 ± 2 °C and a uniform stirring of 100 rpm. At preordained time intervals of 0.5, 1, 2, 4, 8, and 12 h, samples were collected and replenished with fresh release medium. The piperine content was quantified using RP-HPLC method and the graph was plotted betwixt time (hours) and% cumulative drug release [20].

### 2.6. Nasal Permeation Study

The nasal permeation of PIP-SPLopt and PIP suspension (control) were assessed using Franz diffusion cell. Franz diffusion cell of effective permeation area of 2.0 cm^2^ and receiving cell volume of 15 mL was used. The goat nasal mucosal membrane was procured from butcher shop and was prepared and affixed on the receiver compartment. The release media was thronged in the receiver cell and PIP-SPLopt in the donor cell. The constant temperature of 37 ± 2 °C for 24 h with constant stirring at 150 rpm was maintained. At preordained time intervals of 0.5, 1, 2, 4, 8, and 12 h, samples were collected and replenished with fresh release medium and piperine content was quantified using RP-HPLC method [20].

### 2.7. Confocal Laser Scanning Microscopy (CLSM)

The PIP suspension with rhodamine B dye (control) and rhodamine B dye loaded spanlastic formulation were applied to the excised nasal mucosal membrane for 6 h at 37 °C mounted on Franz diffusion cells. After 6 h, to remove the excess amount of dye, nasal mucosa samples were cleansed with distilled water. Then the samples were mounted on the glass slide and sliced into small sections of 6–10 um thickness. Then the slide was espied under CLSM (Leica TC SPE-1lw, Leica microsystem, Heidelberg, Germany) employing argon laser beam (emission at 570 nm and excitation at 488 nm) [20].

### 2.8. Nasal Toxicity Study

The toxicity of the prepared PIP-SPL formulation was scrutinized by performing nasal toxicity study using fresh goat nasal mucosa. The nasal mucosa was stowed in formalin and until a few hours of experiment, preserved in nasal saline. Further it was cut into small sections and treated with a nasal ciliary toxicity agent—isopropyl alcohol (positive control), PIP solution (negative control), and PIP-SPLopt formulation, respectively. The samples were cleaned properly with nasal saline after 2 h of treatment, and then stowed in formalin solution (10%) for histopathological study [20]. The samples were stained with eosin and hematoxylin and observed under optical microscope.

### 2.9. Blood-Brain Distribution Study

Wistar albino rats (100–150 g) were selected and housed into groups of 3 per cage in 12 h light–dark cycle at 25 ± 2 °C after approval from the Institutional Animal Ethics Committee (IAEC) of Jamia Hamdard (Protocol No. 1848). Nine Wistar albino rats were distributed into 3 groups of 3 animals each: Group I as control, Group II treated with PIP-SPL oral formulation (3 mg/mL), and Group III treated with PIP-SPL intranasally (1 mg/mL). The rats were anaesthetized using thiopentone sodium and at 0 (pre-dose), 4, 8, and 24 h; 0.2 mL of blood was collected from tail vein of rats in EDTA-containing tube. Tubes were stowed for 30 min at room temperature and then the plasma was separated from blood through centrifugation (10,000 rpm, 10 min). The plasma samples were separated and stowed at −20 °C. The brains were collected and homogenized. After centrifugation (3000 rpm, 15 min), the supernatants were separated and stowed at −20 °C and the piperine content was analyzed using the RP-HPLC method and Cmax and AUC was calculated [29].

### 2.10. Anticonvulsant Activity

Wistar albino rats (100–150 g) were acquired from Central Animal House Facility, Jamia Hamdard, New Delhi, India and kept under standard laboratory conditions (12 h-light dark cycle, 25 ± 2 °C) after approval from the Institutional Animal ethics committee (IAEC Approval No. 1848). The study was carried out to determine the anticonvulsant activity of PIP-SPL formulation. The rats in groups of 6 were distributed into five groups: Group P was treated as negative control (toxic group), Group Q was treated with standard diazepam (4 mg/mL), Group R was treated with PIP-SPL formulation intranasally (1 mg/mL), Group S was treated with PIP intranasally (3 mg/mL), and Group T was treated as positive control (normal control).

#### 2.10.1. Pentylenetetrazole (PTZ)-Induced Seizure

PTZ (85 mg/kg) was injected intraperitoneally to rats 30 min after administration of PIP formulation, PIP suspension and diazepam. The rats were monitored for 1 h following the PTZ injection and the latency time of the onset of clonic, myoclonic, and tonic convulsions were noted. Additionally, number of deaths per group were noted up to 24 h after injection of PTZ [30].

#### 2.10.2. Effect on Neuromuscular Coordination

The effect of PIP-SPL formulation on coordinated motor movements was scrutinized using the rotarod test. Before 24 h of the test, the rats were trained on the rotarod for 60 s. On the next day, before and 30 min after administration of PIP-SPL formulation, PIP, and diazepam, the rats were tested on rotarod. The stay time for each rat on rotarod was noted for a maximum of 300 s [31].

#### 2.10.3. Biochemical Estimation

The animals were sacrificed, and their brains were removed and homogenized in phosphate buffer using homogenizer. After centrifugation for 10 min at 3000 rpm, clear supernatant was obtained and used for malondialdehyde, reduced glutathione (GSH), superoxide dismutase (SOD), and catalase (CAT) estimation [30].

#### 2.10.4. Histopathological Study

The hippocampus of brains were collected after sacrificing the rats and stowed in formalin solution (10%) and stained with dyes (eosin and hematoxylin) for histopathological examination [30].

### 2.11. Statistical Analysis

The results were represented in the form of mean ± standard error of the mean. The data acquired from PTZ experiments were analyzed using “Dunnett *t* test” and “one-way analysis of variance”. The data acquired from the rotarod experiment were analyzed using “analysis of covariance”. A *p* value of less than 0.05 was contemplated statistically significant.

## 3. Results and Discussion

### 3.1. Optimization of PIP-SPL by BBD

**Figure 1:**
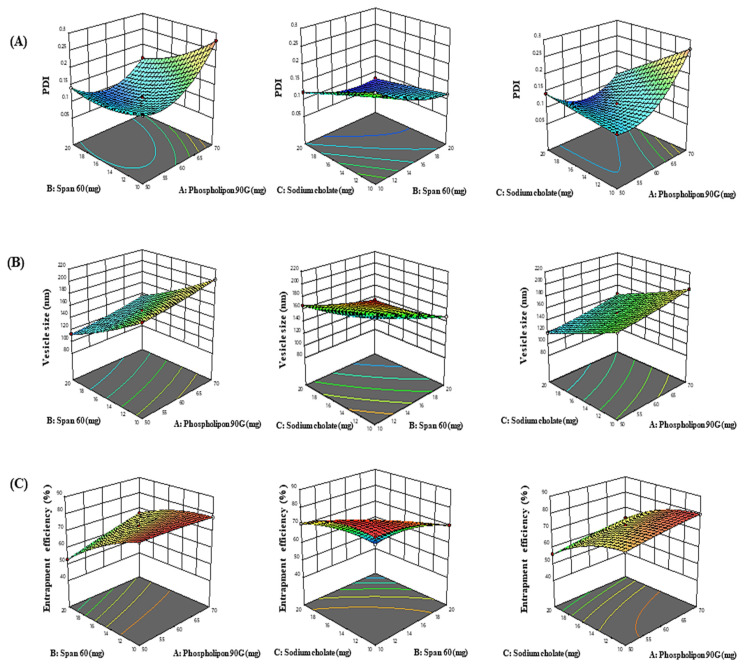
3D-response graphs representing the effect of independent variables on (**A**) PDI, (**B**) vesicle size, and (**C**) entrapment efficiency.

**Figure 2:**
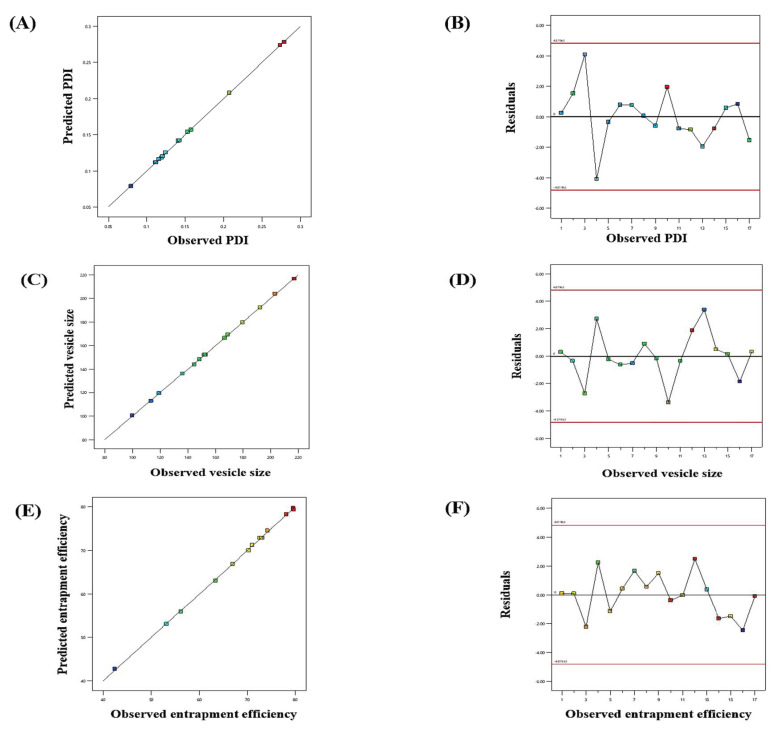
Linear correlation plots (**A**,**C**,**E**) between actual and predicted values and the corresponding residual plots (**B**,**D**,**F**) for various responses.

The impact of adopted parameters (phospholipon 90G, span 60, and sodium cholate) on PDI, entrapment efficiency, and vesicle size of PIP-loaded spanlastics are evinced by the 3D-response diagrams illustrated in Figure 1 and the corresponding residual plots for adopted responses and the linear relationship betwixt predicted and experimental values (engendered by BBD) are represented in Figure 2.

#### 3.1.1. Response (1): Effect of Independent Variables on PDI

The PDI of all 17 runs were reckoned to be betwixt 0.0792 and 0.2789 (Table 2).

PDI = + 0.1115 + 0.0348 A − 0.0334 B − 0.0312 C − 0.0273 AB − 0.0431 AC + 0.0126 BC +0.0525 A^2^ + 0.0185 B^2^ + 0.0007 C^2^

From the above polynomial equation, it was signified that the phospholipon 90G has a positive effect on PDI. On increasing concentration of phospholipon 90G (50 to 70 mg), the PDI increased from 0.1192 ± 0.003 to 0.2734 ±0.007, 0.1241 ± 0.003 to 0.1423 ± 0.012, 0.1408 ± 0.005 to 0.1575 ± 0.004, and 0.1532 ± 0.008 to 0.2789 ± 0.012 as observed in formulations 3 and 14, 4 and 7, 13 and 2, and 17 and 10, respectively. In contrast, span 60 and sodium cholate have negative effect on PDI. Increasing concentration of span 60 from 10 to 20 mg leads to decreased PDI from 0.2789 ± 0.003 to 0.1575 ± 0.005, 0.2074 ± 0.004 to 0.1156 ± 0.002, 0.1207 ± 0.012 to 0.0792 ± 0.004, and 0.1532 ± 0.007 to 0.1408 ± 0.003 as observed in formulations 10 and 2, 12 and 9, 15 and 16, and 17 and 13, respectively. Similarly, increasing sodium cholate concentration from 10 to 20 mg results in decreased PDI from 0.1423 ± 0.012 to 0.1192 ± 0.003, 0.1156 ± 0.003 to 0.0792 ± 0.002, 0.2074 ± 0.008 to 0.1207 ± 0.007, and 0.2734 ± 0.007 to 0.1241 ± 0.004 as observed in formulations 7 and 3, 9 and 16, 12 and 15, and 14 and 4, respectively.

#### 3.1.2. Response (2): Effect of Independent Variables on Vesicle Size

The vesicle size of all 17 runs were reckoned to be betwixt 99.92 and 217.12 (Table 2).

Vesicle size = + 152.23 + 11.92 A – 33.60 B – 24.54 C – 0.2150 AB + 0.4700 AC + 0.5575 BC + 2.11 A^2^ + 3.78 B^2^ + 1.95 C^2^

From the experimental data, it was signified that the phospholipon 90G has a positive effect on vesicle size. By increasing phospholipon 90G concentration from 50 to 70 mg, the vesicle size increases from 168.81 ± 4.09 to 192.43 ± 4.03 nm, 119.21 ± 3.92 to 144.71 ± 2.98 nm, 113.43 ± 4.36 to 136.12 ± 2.32 nm, and 179.69 ± 3.07 to 203.24 ± 4.26 nm as observed in formulations 3 and 14, 7 and 4, 13 and 2, and 17 and 10, respectively. This could be attributable to the fact that increase in phospholipon 90G results in prominent increase of vesicle size, wherein span 60 and sodium cholate exhibited the negative impact on vesicle size. By increasing span 60 concentration from 10 to 20 mg, the vesicle size is reduced from 203.24 ± 3.92 to 136.12 ± 4.36 nm, 217.12 ± 4.23 to 148.29 ± 2.09 nm, 166.52 ± 2.98 to 99.92 ± 2.32 nm, and 179.69± 4.35 to 113.43 ± 4.67 nm as observed in formulations 10 and 2, 12 and 9, 15 and 16, and 17 and 13, respectively. Similarly, by increasing sodium cholate concentration from (10 to 20 mg), the vesicle size is reduced from 168.81 ± 4.26 to 119.21 ± 4.03 nm, 148.29 ± 4.67 to 99.92 ± 2.09 nm, 217.12 ± 3.07 to 166.52 ± 4.09 nm, and 192.43 ± 4.35 to 144.71 ± 4.23 nm as observed in formulations 3 and 7, 9 and 16, 12 and 15, and 14 and 4, respectively. The decrease in vesicle size by sodium cholate and span 60 could be due to the disruption of bilayer structure of cellular membrane beyond their certain concentration.

#### 3.1.3. Response (3): Effect of Independent Variables on Entrapment Efficiency

From experimental data, it was observed that independent variables have significant effect on entrapment efficiency (EE) and the EE of all 17 runs were found to be betwixt 42.41 and 79.62% (Table 2).

Entrapment efficiency = + 72.90 + 3.11 A − 9.45 B − 8.84 C + 3.80 AB + 0.4725 AC − 4.82 BC − 0.5155 A^2^ − 2.96 B^2^ − 4.10 C^2^

From the above polynomial equation, it was signified that phospholipon 90G has a positive effect on entrapment efficiency, wherein span 60 and sodium cholate have negative effect on entrapment efficiency. It was observed that an increment in phospholipon 90G concentration (50 to 70 mg) improved the entrapment efficiency from 74.18 ± 1.34 to 79.51 ± 2.98%, 56.1 ± 1.72 to 63.32 ± 1.23%, 53.12 ± 2.09 to 66.89 ± 1.62%, and 78.12 ± 1.92 to 79.54 ± 1.68% as observed in formulations 3 and 14, 7 and 4, 13 and 2, and 10 and 17, respectively. This could be due to the expansion of bilayer domain dimension resulting from the formation of a greater number of TN vesicles, which provides more space for PIP entrapment in TN vesicles.

From the experimental data, it can be signified that the increase in span concentration (10 to 20 mg) results in decreased entrapment efficiency of PIP in TN vesicles from 78.12 ± 1.23 to 76.89 ± 1.72%, 79.62 ± 1.62 to 70.26 ± 1.76%, 71.03 ± 1.92 to 42.41 ± 1.23%, and 79.54 ± 1.98 to 53.12 ± 1.87% as observed in formulations 10 and 2, 12 and 9, 15 and 16, and 17 and 13, respectively. Similarly, increase in sodium cholate concentration (10 to 20 mg) results in decreased entrapment efficiency from 74.18 ± 1.62 to 56.1 ± 2.98%, 70.26 ± 1.87 to 42.41 ± 1.76%, 79.62 ± 2.09 to 71.03 ± 1.34%, and 79.51 ± 1.98 to 63.32 ± 1.62% as observed in formulations 3 and 7, 9 and 16, 12 and 15, and 14 and 4, respectively. This could be due to the fact that beyond a certain concentration, span 60 and sodium cholate disrupts the vesicular bilayered membrane structure, which leads to loss of the drug from the TN vesicles.

Based on the above experimental results, optimized formulation was prepared with phospholipon 90G (60 mg), span 60 (15 mg), and sodium cholate (15 mg) as per the formula generated by the point prediction method and further evaluated for PDI, entrapment efficiency and vesicle size. The PIP-SPLopt exhibited the vesicle size of 152.4 ± 2.98 nm, entrapment efficiency of 72.93 ± 2.09% and PDI of 0.1118 ± 0.009, which were in proximity with the predicted values of PDI of 0.1115, vesicle size of 152.23 nm, and entrapment efficiency of 72.90% engendered by the Box–Behnken design.

### 3.2. Characterization

**Figure 3:**
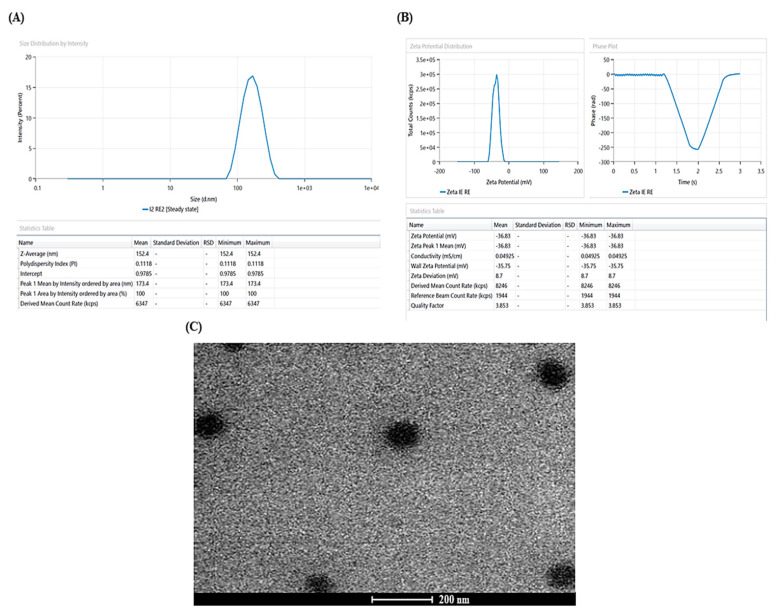
(**A**) Vesicle size distribution of PIP-SPLopt formulation, (**B**) zeta-potential of PIP-SPLopt formulation, and (**C**) transmission electron microscopy of PIP−SPLopt formulation.

Average vesicle size and PDI of PIP-SPLopt was experimentally reckoned to be 152.4 nm and 0.1118, respectively (Figure 3A), with entrapment efficiency of 72.93%, wherein the predicted values of average vesicle size and PDI were 152.23 nm and 0.1115 with an entrapment efficiency of 72.90%. The experimentally observed values of all responses were in proximity with the predicted values confirming the legitimacy and consistency of the model. Furthermore, the zeta potential of PIP-SPLopt was reckoned to be −36.83 mV (Figure 3B).

### 3.3. Spanlastic Morphology

The TEM analysis of PIP-SPLopt formulation unveiled that the prepared vesicles were spherical in shape with a well-defined sealed structure and uniform size distribution (Figure 3C).

### 3.4. In Vitro Drug Release Study

**Figure 4:**
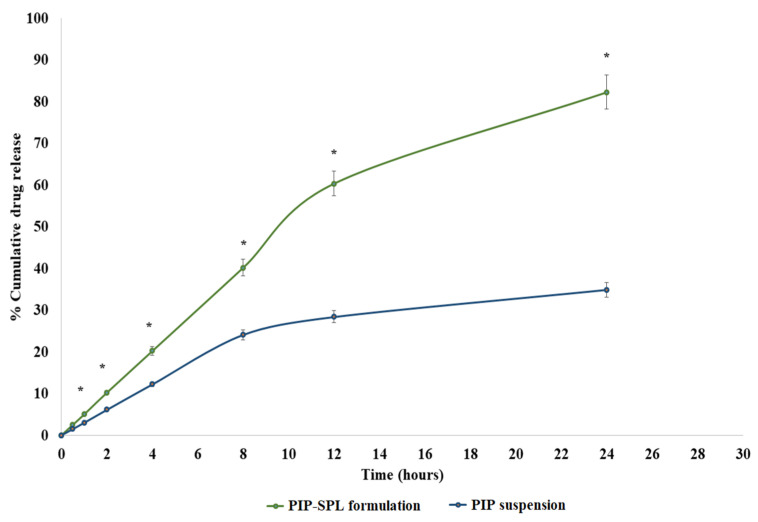
Comparative in vitro drug release profile of PIP suspension and PIP-SPLopt formulation. *: statistically significant at *p* ≤ 0.05.

The in vitro release of piperine from PIP suspension through dialysis membrane was reckoned to be 34.91%, wherein the optimized PIP-SPLopt formulation presented an 82.32% release of piperine through dialysis membrane (Figure 4). At each time point, a significant drug release was achieved. In comparison to pure PIP, the PIP-SPLopt formulation exhibited a delayed drug release. Spanlastic is able to limit drug release because PIP must transverse the brain lipid bilayer and is able to diffuse slowly. The graph shows that the medication is released rapidly during the first four hours and then at a reduced rate over the next 24 h. This sort of releasing behavior is optimal for improving treatment effectiveness. Initial rapid release aids in achieving therapeutic concentration but extended gradual release improves therapeutic efficacy. Data acquired from an in vitro drug release experiment was fitted into diverse mathematical kinetics models (first-order, zero-order, Korsmeyer–Peppas, and Higuchi kinetics models), out of which the Kosmeyer–Peppas model revealed the maximum R^2^ value illustrated in Table 3. Ergo, it can be stated that the release of piperine from PIP-SPLopt formulation follows a Kosmeyer–Peppas diffusion mechanism.

### 3.5. Nasal Permeation Study

**Figure 5:**
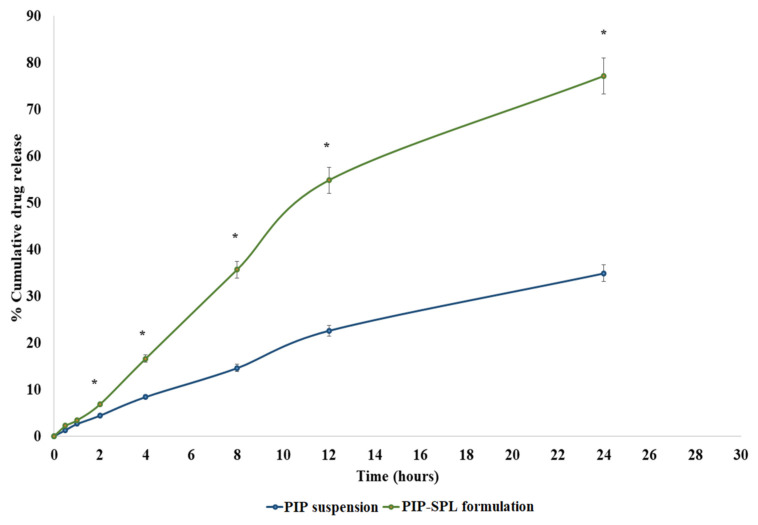
Comparative in vitro skin permeation study of PIP suspension and PIP-SPLopt formulation across goat nasal mucosa membrane. *: statistically significant at *p* ≤ 0.05.

The nasal permeation study revealed the permeation of 34.89% of piperine from PIP suspension with flux value of 1.95 µg/cm^2^/h, wherein the PIP-SPLopt formulation exhibited permeation of 77.12% piperine through the nasal mucosa with flux value of 4.31 µg/cm^2^/h. The presence of span 60 and edge activator aids in the solubilization of lipophilic PIP and yields 2.21-fold greater permeation of PIP from PIP-SPLopt nanovesicles than PIP suspension (Figure 5).

### 3.6. Confocal Laser Scanning Microscopy

**Figure 6:**
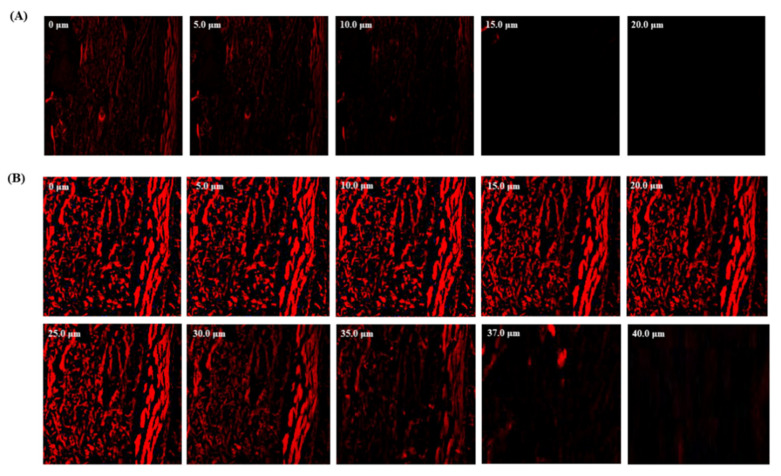
CLSM images in optical cross section perpendicular to goat nasal mucosa membrane (**A**) treated with PIP suspension and (**B**) treated with PIP-SPLopt formulation.

The CLSM analysis revealed that the PIP-SPL formulation penetrated deeper into the layer of nasal mucosa (up to 32 µm) in comparison with the PIP suspension (control), which was confined to a depth of 10 um perse (Figure 6). The higher fluorescence intensity by PIP-SPLopt formulation unveiled that the PIP was uniformly distributed throughout the deeper layers of nasal membrane to a great extent in comparison to PIP suspension, which also confirms the higher permeation.

### 3.7. Nasal Toxicity Study

**Figure 7:**
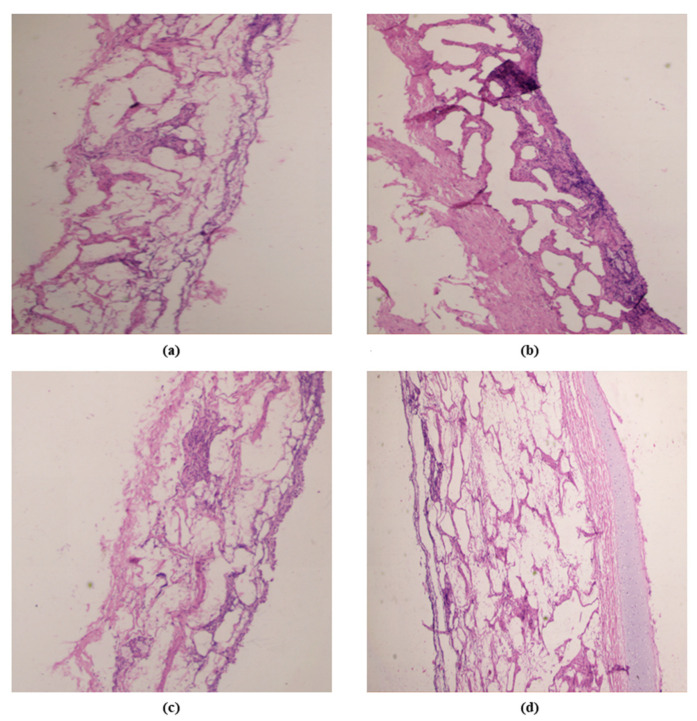
Histopathological optical micrograph structure of goat nasal mucosa treated with (**a**) nasal saline buffer pH 6.4, (**b**) isopropyl alcohol, (**c**) PIP suspension, and (**d**) PIP-SPL formulation (20× magnification).

The nasal toxicity study shown in Figure 7 revealed that the nasal saline-treated group (negative control) exhibited epithelial layer lining underneath connective tissue with glandular structure and blood vessels; the isopropyl-treated group (positive control) showed connective tissue and epithelial layer damage, whereas PIP-SPL formulation exhibited unremarkable change in epithelial layer and connective tissue structure, but PIP suspension showed epithelial cell focal sloughing. Overall study unveiled that PIP-SPL formulation was reckoned to be safer for delivering PIP into the brain through the intranasal route.

### 3.8. Blood–Brain Distribution Study

**Figure 8:**
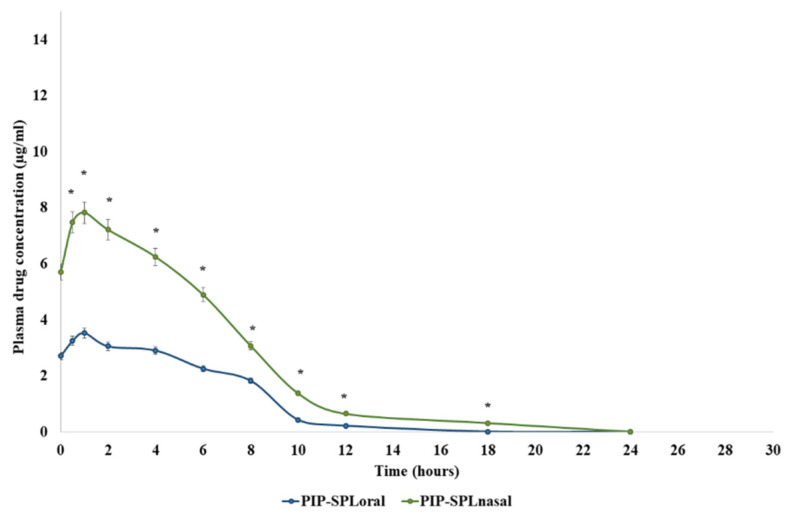
Plasma drug concentration profiles of piperine after oral and intranasal delivery. *: statistically significant at *p* ≤ 0.01.

**Figure 9:**
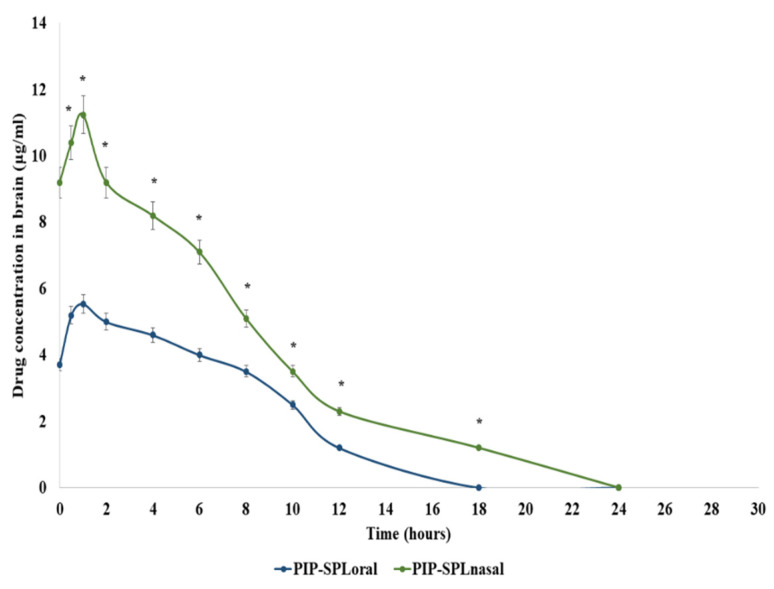
Drug concentration profiles of piperine in brain after oral and intranasal delivery. *: Statistically significant at *p* ≤ 0.01.

Blood–brain distribution studies were performed on rats to assess the in vivo behavior of PIP-SPL formulation in the blood and brain. The concentration of PIP in the plasma and brain after oral and intranasal administration of PIP-SPL formulation was measured by HPLC (HPLC graphs are shown in supplementary materials) using the reported method, and the results are illustrated in Figure 8 and Figure 9 and Table 4. C_max_ and AUC_0–24h_ of the PIP-SPL formulation in blood and brain after oral administration were 3.46 ± 1.91 µg/mL and 22.68 ±9.76 µg·h/mL and 5.34 ± 1.12 µg/mL and 49.68 ±5.63 µg·h/mL, respectively, which were significantly lower than those of (*p* < 0.01) the intranasally applied PIP-TN formulation (7.72 ± 0.51 µg/mL and 38.96 ± 7.90 µg·h/mL and 10.24 ± 1.21 µg/mL and 61.76 ±2.36 µg·h/mL, respectively) revealing that absorption of PIP was potentially increased on intranasal application of PIP-TN formulation (Figure 8 and Figure 9).

### 3.9. Anticonvulsant Activity

#### 3.9.1. Effect of PIP-SPL Formulation on PTZ-Induced Seizures in Rats

**Figure 10:**
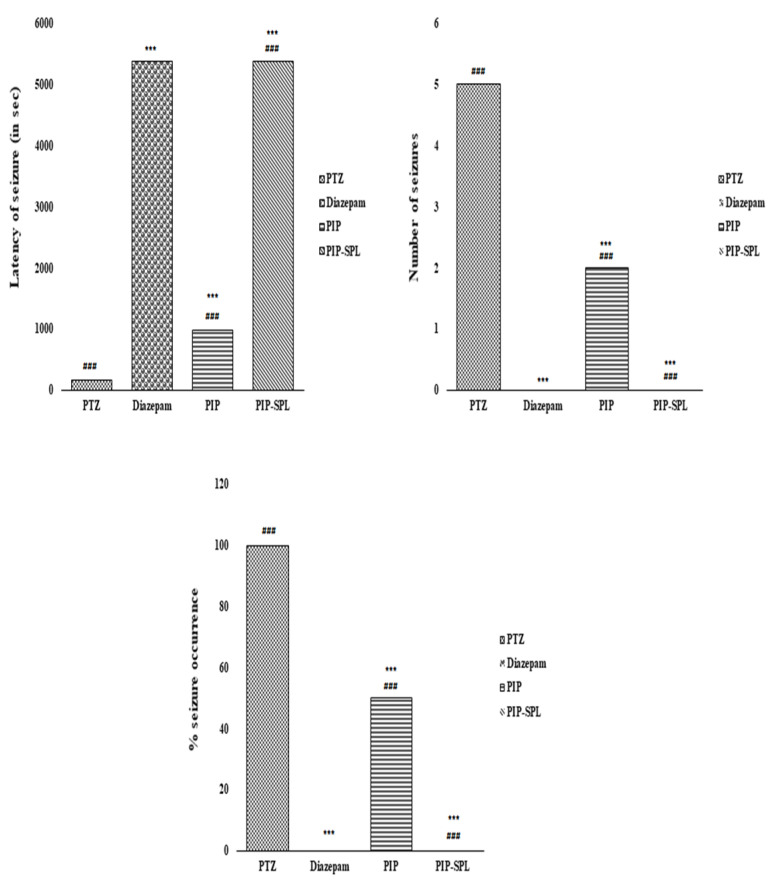
Effect of different treatments on latency of seizures, no. of seizures, and % seizure occurrence (*** indicates that the comparison was made between normal vs. other groups, whereas ### indicates that the comparison was made between control and other groups).

Rats were observed for seizures after PTZ administration (85 mg/kg i.p.). Seizures were initiated within 10–25 min of PTZ administration and proceeded to the advanced stage (stages 4 and 5). In the present study, rats pretreated with PIP-SPL showed a significant decrease in % kindling and seizure score and augmented the onset time of seizures in comparison to PTZ (*p* < 0.05). The cutoff time of 90 min was taken for all groups, which implies that no seizures occurred till the cutoff time. PIP and PIP-SPL both significantly reduced the seizure score (*p* < 0.01) compared to PTZ. The PIP-SPL group protected 100% of rats from the occurrence of PTZ induced seizure compared to PIP, wherein 50% of animals showed occurrence of seizures (Figure 10 and Table 5). A similar study was conducted by Kola et al., and they observed that the naringin at 80 mg/kg dose showed more protection against PTZ-induced seizure and reduction in kindling process development and % of animals kindled [30]. Yu et al. also observed that rhein can reverse the PTZ-induced epilepsy by inhibition of TLR4 and can be used in neural inflammation due to its neuroprotective effect [31].

#### 3.9.2. Effect of PIP-SPL Formulation on Neuromuscular Coordination in Rats

The independent t-test revealed that diazepam and PIP-SPL treated groups caused a significant decrease in the stay time of rats (in no. of seconds) on rotarod in comparison with the control group. The group treated with PIP suspension, PIP-SPL, and diazepam showed an increased stay time of the rats on rotarod compared to the PTZ-treated group (*p* < 0.05). However, the PIP-SPL group showed higher neuromuscular coordination than diazepam, which could be attributable to higher permeation of PIP-SPL formulation via intranasal route (Table 6). Yu et al. performed the similar study and observed that rhein can minimize the neurological impairment caused by PTZ-induced epilepsy [31]. Similarly, PIP-SPL can also minimize neurological impairment.

#### 3.9.3. Effect of PIP-SPL on PTZ Kindling-Induced Oxidative Biomarkers on the Brain

**Figure 11:**
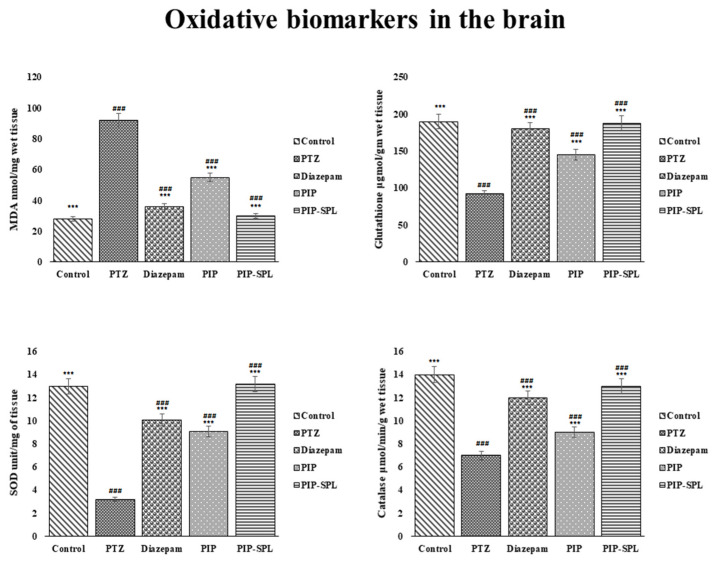
Effect of different treatments on oxidative stress markers of brain (*** indicates that the comparison was made between normal vs. other groups, whereas ### indicates that the comparison was made between control and other groups).

The biochemical estimations of oxidative biomarkers (GSH, SOD, CAT, and MDA) were performed, and from the experimental results, it was observed that CAT, GSH, and SOD levels dwindled and MDA level was elevated in the brains of PTZ-induced rats compared to the control group, resulting in degenerative and oxidative stress effects due to oxidative free radicals accretion wherein PIP-SPL formulation, PIP suspension, and diazepam exhibited significant reduction in MDA level and elevation in CAT, GSH, and SOD levels but lesser in comparison with the control group (Table 7). Moreover, the PIP-SPL formulation and standard diazepam showed nearly the same CAT and SOD activity but not higher than that of the toxic group (*p* < 0.05), implying good antiepileptic potential (Figure 11).

The PIP-SPL formulation exhibited more improvement in oxidative biomarkers than standard diazepam by restoring the oxidative enzyme levels to normal, showing its antioxidant effect and neuroprotective effect, which could be attributable to the higher permeation of PIP-SPL formulation in the brain via the intranasal route (Figure 11). A similar observation was observed by Saleha et al., who observed an elevation in oxidative stress enzymes on administration of ketamine and a decrease in oxidative stress enzymes on administration of paliperidone-loaded lipid nanoconstructs via brain delivery due to its antioxidant and neuroprotective effect [32]. A similar study was conducted by Shafaei et al., who observed that lipid peroxidation increases upon administration of cadmium and decreases when Anethum graveolens seed oil nanoemulsion was administered by showing its antioxidant effect [33].

#### 3.9.4. Histological Examination

**Figure 12:**
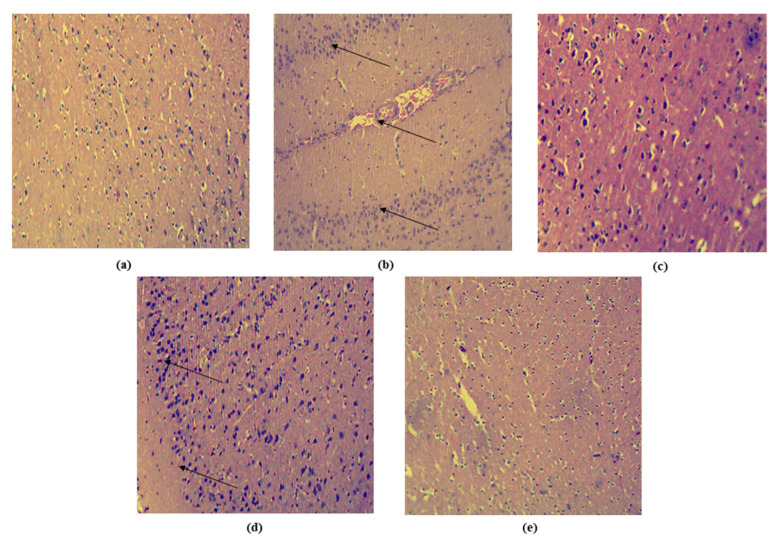
Histopathological images of brain hippocampi stained with hematoxylin and eosin under different treatments: (**a**) control, (**b**) PTZ (toxic control), (**c**) diazepam, (**d**) PIP suspension, and (**e**) PIP-SPL formulation. Arrows show neurodegeneration and neuronal cell death (10× magnification).

A histological examination of the hippocampus of the brains was performed, which revealed that the hippocampus of the rats in the toxic group (PTZ-treated group) exhibited neurodegeneration and severe neuronal cell damage. The rats treated with standard diazepam and PIP suspension showed occasional neuronal cell damage with less neurodegeneration. In contrast, the group treated with PIP-SPL formulation exhibited the most extensive protection from neurodegeneration, with no neuronal cell damage among the other groups (Figure 12).

## 4. Conclusions

The current research comprehends the development and optimization of PIP-loaded SPL formulation by utilizing BBD, which engendered better anti-epileptic activity via the intranasal route. The elite PIP-SPL formulation manifested a nano size of 152.4 nm, EE of 72.93%, with a PDI of 0.1118. The CLSM study unveiled that the developed PIP-SPL formulation has greater permeation of PIP across the nasal mucosa in comparison with PIP suspension and a greater in vitro release withal. The blood–brain distribution study demonstrated significant levels of PIP in the blood and brain via the intranasal route in comparison with oral administration. The in vivo study unveiled that the PIP-SPL formulation manifested an enhanced anticonvulsant effect compared to the standard diazepam based on seizure activity, neuromuscular coordination, and oxidative biomarker activity. The outcomes corroborate that the prepared SPL vesicle formulation is a treasured carrier in PIP intranasal delivery for the management of epilepsy.

## Figures and Tables

**Table 1:** Variables and their levels.

Variables	Levels		
	Low (−1)	Medium (0)	High (+1)
Independent variables			
A: phospholipon 90G (mg)	50	60	70
B: span 60 (mg)	10	15	20
C: Sodium cholate (mg)	10	15	20
Dependent Variable			
Y_1_: PDI Y_2_: Vesicle size (nm) Y_3_: Entrapment efficiency (%)			

**Table 2:** Box–Behnken experimental design with measured responses.

Formulation	Independent Variables			Dependent Variables		
	A	B	C	Y_1_	Y_2_	Y_3_
1	60	15	15	0.1118 ± 0.007	152.4 ± 4.09	72.93 ± 1.34
2	70	20	15	0.1575 ± 0.003	136.12 ± 3.92	66.89 ± 1.68
3	50	15	10	0.1192 ± 0.009	168.81 ± 2.98	74.18 ± 2.09
4	70	15	20	0.1241 ± 0.009	144.71 ± 2.98	63.32 ± 2.09
5	60	15	15	0.1112 ± 0.003	152.1 ± 3.92	72.56 ± 1.68
6	60	15	15	0.1123 ± 0.003	151.9 ± 3.92	73.03 ± 1.68
7	50	15	20	0.1423 ± 0.003	119.21 ± 4.03	56.1 ± 2.98
8	60	15	15	0.1116 ± 0.012	152.7 ± 4.26	73.07 ± 1.62
9	60	20	10	0.1156 ± 0.005	148.29 ± 4.36	70.26 ± 1.72
10	70	10	15	0.2789 ± 0.003	203.24 ± 4.67	78.12 ± 1.87
11	60	15	15	0.1108 ± 0.009	152.04 ± 2.98	72.89 ± 2.09
12	60	10	10	0.2074 ± 0.004	217.12 ± 4.23	79.62 ± 1.62
13	50	20	15	0.1408 ± 0.003	113.43 ± 3.92	53.12 ± 1.68
14	70	15	10	0.2734 ± 0.002	192.43 ± 2.09	79.51 ± 1.76
15	60	10	20	0.1207 ± 0.004	166.52 ± 2.32	71.03 ± 1.23
16	60	20	20	0.0792 ± 0.008	99.92 ± 3.07	42.41 ± 2.09
17	50	10	15	0.1532 ± 0.003	176.69 ± 3.92	79.54 ± 1.68
**Quadratic model**		**R^2^**	**Adjusted R^2^**	**Predicted R^2^**	**S.D.**	**%CV**
**Response (Y_1_)**		0.9998	0.9997	0.9980	0.0010	0.7202
**Response (Y_2_)**		0.9998	0.9996	0.9979	0.5750	0.3688
**Response (Y_3_)**		0.9995	0.9990	0.9941	0.3316	0.4783

**Table 3:** Invitro drug release kinetics with correlation values.

Release Kinetics	R^2^	Equation	X-Axis	Y-Axis
Korsmeyer–Peppas	0.989	M_t_/M∞ = K_tn_	Log fraction of drug released	Log time
Higuchi	0.952	M_t_ = M_0_ + k_h_t_1/2_	Fraction of drug released	√time
Zero-order release	0.971	M_t_ = M_0_ + k_0_ t	Fraction of drug released	time
First-order release	0.976	ln M_t_ = ln M_0_ + k_1_ t	Log % drug remaining	time

**Table 4:** Pharmacokinetic parameters.

**Pharmacokinetic Parameters**	**PIP-SPL Oral (In Blood)**	**PIP-SPL Nasal (In Blood)**
C_max_	3.46 ± 1.91 ^a^ µg/mL	7.72 ± 0.51 ^b^ µg/mL
T_max_	1 ^a^ h	1 ^b^ h
T_1/2_	9 ^a^ h	12 ^b^ h
AUC_0–24h_	22.68 ± 9.76 ^a^ µg·h/mL	38.96 ± 7.90 ^b^ µg·h/mL
**Pharmacokinetic Parameters**	**PIP-SPL Oral (In Brain)**	**PIP-SPL Nasal (In Brain)**
C_max_	5.34 ± 1.12 ^a^ µg/mL	10.24 ± 1.21 ^b^ µg/mL
T_max_	1 ^a^ h	1 ^b^ h
T_1/2_	9 ^a^ h	12 ^b^ h
AUC_0–24h_	49.68 ± 5.63 ^a^ µg·h/mL	61.76 ± 2.36 ^b^ µg·h/mL

Values with different superscript letters in the same row are significantly different (*p* ≤ 0.01).

**Table 5:** Effect of different treatments on seizure activity.

	Latency of Myoclonic Jerks (s)	Latency of GTSs (s)	Duration of GTSs (min)	% of Animals Kindled	% Protection	No. of Seizures	Seizure Score
PTZ	57.6 ± 2.92 ^a^	252.23 ± 3.07 ^a^	35.33 ± 2.01 ^a^	100 ^a^ %	0 ^a^ %	5 ^a^	5 ^a^
Diazepam	Not observed	Not observed	Not observed	0 ^b^ %	100 ^b^ %	0 ^b^	0 ^b^
PIP suspension	474 ± 4.75 ^c^	971.65 ± 6.23 ^c^	17.23 ± 1.23 ^c^	50 ^c^ %	50 ^c^ %	2 ^c^	2 ^c^
PIP-SPL formulation	1263 ± 9.23 ^d^	Not observed	Not observed	0 ^d^ %	100 ^d^ %	0 ^d^	0 ^d^

Values with different superscript letters in the same row are significantly different (*p* ≤ 0.05).

**Table 6:** Effect of different treatments on neuromuscular coordination.

	Stay Time for Each Rat on Rotarod (in Seconds)
**Control**	500 ± 5 ^a^ seconds
**PTZ**	28 ± 2 ^b^ seconds
**Diazepam**	412 ± 3 ^c^ seconds
**PIP suspension**	376 ± 2 ^d^ seconds
**PIP-SPL formulation**	452 ± 4 ^e^ seconds

Values with different superscript letters in the same row are significantly different (*p* ≤ 0.05).

**Table 7:** Effect of different treatments on oxidative stress markers of the brain.

	MDA (nmol/mg Wet Tissue)	Glutathione (ugmol/gm Wet Tissue)	Catalase (umol/min/g Wet Tissue)	SOD (Unit/mg of Tissue)
Control	28 ± 2.01 ^a^	190 ± 5.76 ^b^	14 ± 1.06 ^c^	13 ± 0.98 ^d^
PTZ	92 ± 3.92 ^a^	92 ± 3.07 ^b^	7 ± 0.21 ^c^	3.2 ± 0.06 ^d^
Diazepam	36 ± 1.96 ^a^	180 ± 4.21 ^b^	12 ± 0.96 ^c^	10.1 ± 1.23 ^d^
PIP suspension	55 ± 1.75 ^a^	145 ± 3.23 ^b^	9 ± 1.23 ^c^	9.1 ± 0.92 ^d^
PIP-SPL formulation	30 ± 2.23 ^a^	188 ± 4.76 ^b^	13 ± 1.87 ^c^	13.2 ± 1.07 ^d^

Values with different superscript letters in the same row are significantly different (*p* ≤ 0.05).

## Data Availability

This study did not report any data (all the data is included in the current manuscript).

## Supplementary Materials

The following supporting information can be downloaded at: https://www.mdpi.com/article/10.3390/pharmaceutics15020641/s1. Figure S1: HPLC Chromatogram of plasma blank; Figure S2: HPLC Chromatogram of Standard piperine in plasma; Figure S3: HPLC Chromatogram of PIP-SPL formulation in plasma; Figure S4: HPLC Chromatogram of brain blank; Figure S5: HPLC Chromatogram of Standard piperine in brain; Figure S6: HPLC Chromatogram of PIP-SPL formulation in plasma; Figure S7: HPLC chromatogram of standard piperine (for encapsulation efficiency); Figure S8: HPLC chromatogram of PIP-SPLopt formulation (for encapsulation efficiency).
