# Supplementary material for: Spanlastics as a Potential Approach for Enhancing the Nose-To-Brain Delivery of Piperine: In Vitro Prospect and In Vivo Therapeutic Efficacy for the Management of Epilepsy
Source: Pharmaceutics. 2023 Feb 14;15(2):641. doi: 10.3390/pharmaceutics15020641 (PMC9959896; doi:10.3390/pharmaceutics15020641)
Supplement: Supplementary file 1 [file pharmaceutics-15-00641-s001.zip › pharmaceutics-2119950-supplementary.pdf]

Supplementary material for Blood-brain distribution study:

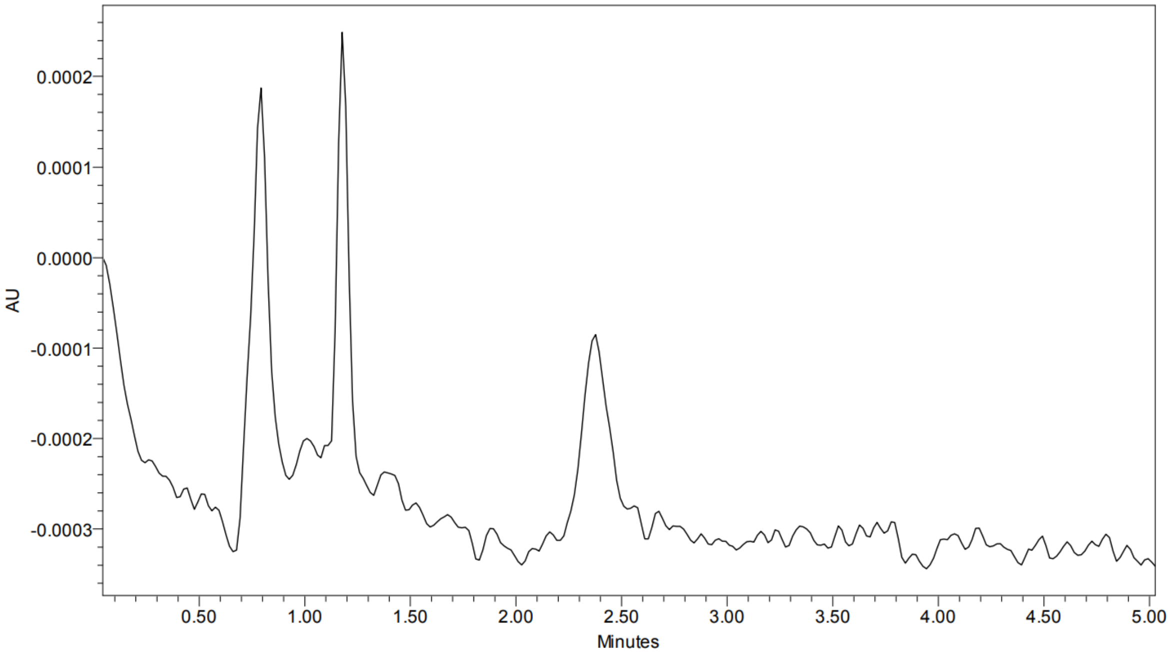

Figure S1: HPLC Chromatogram of plasma blank

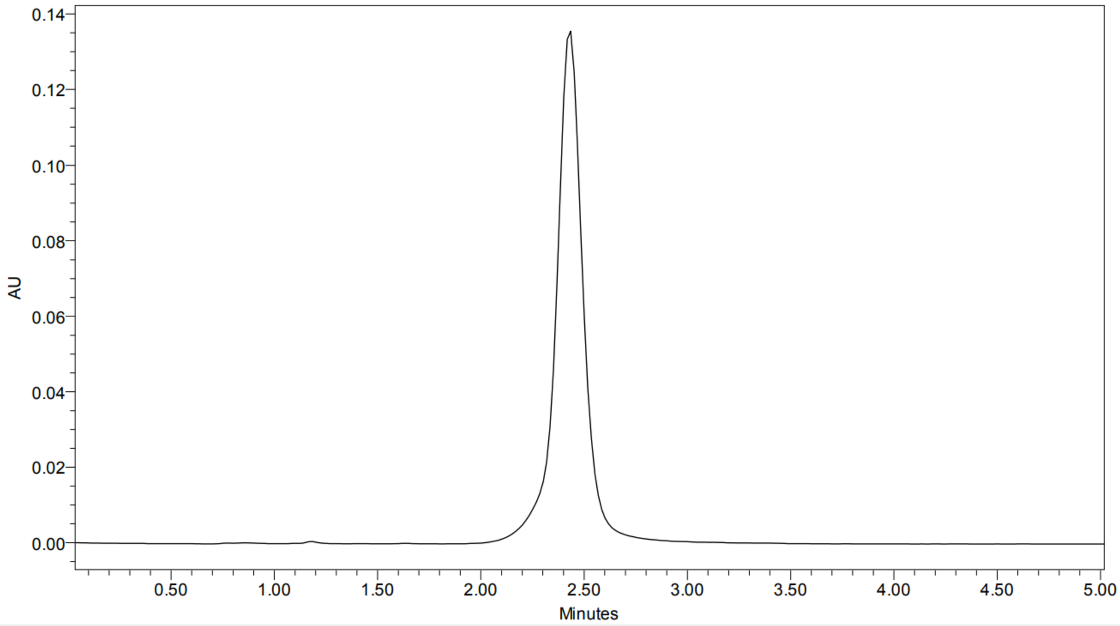

Figure S2: HPLC Chromatogram of Standard piperine in plasma

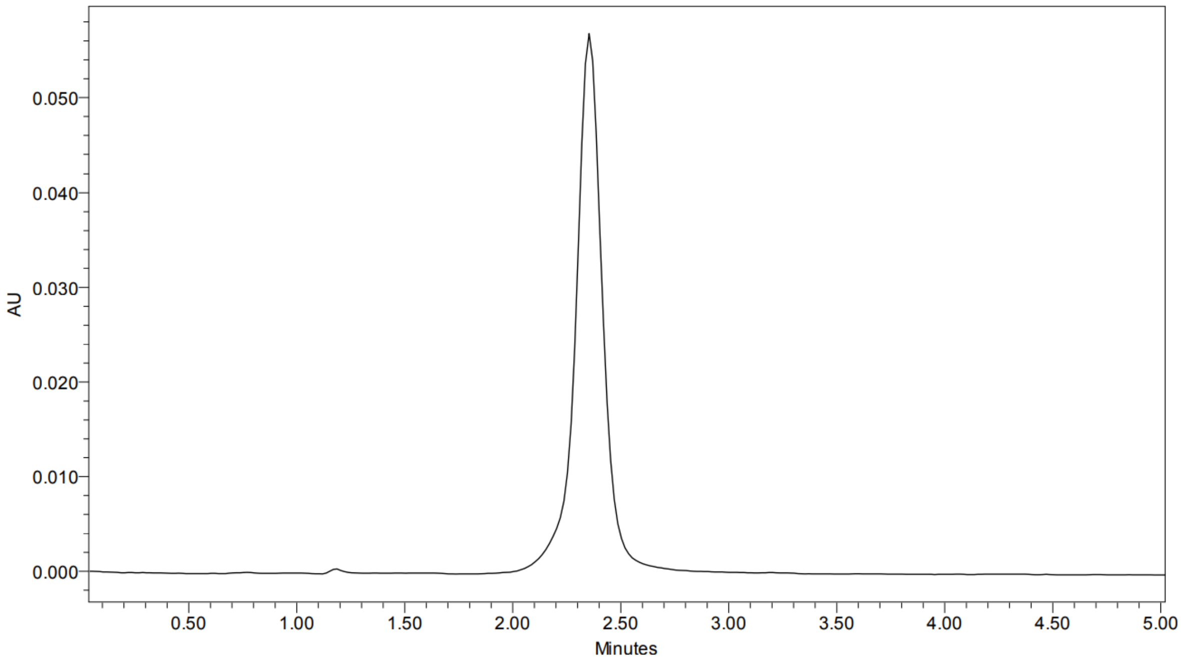

Figure S3: HPLC Chromatogram of PIP-SPL formulation in plasma

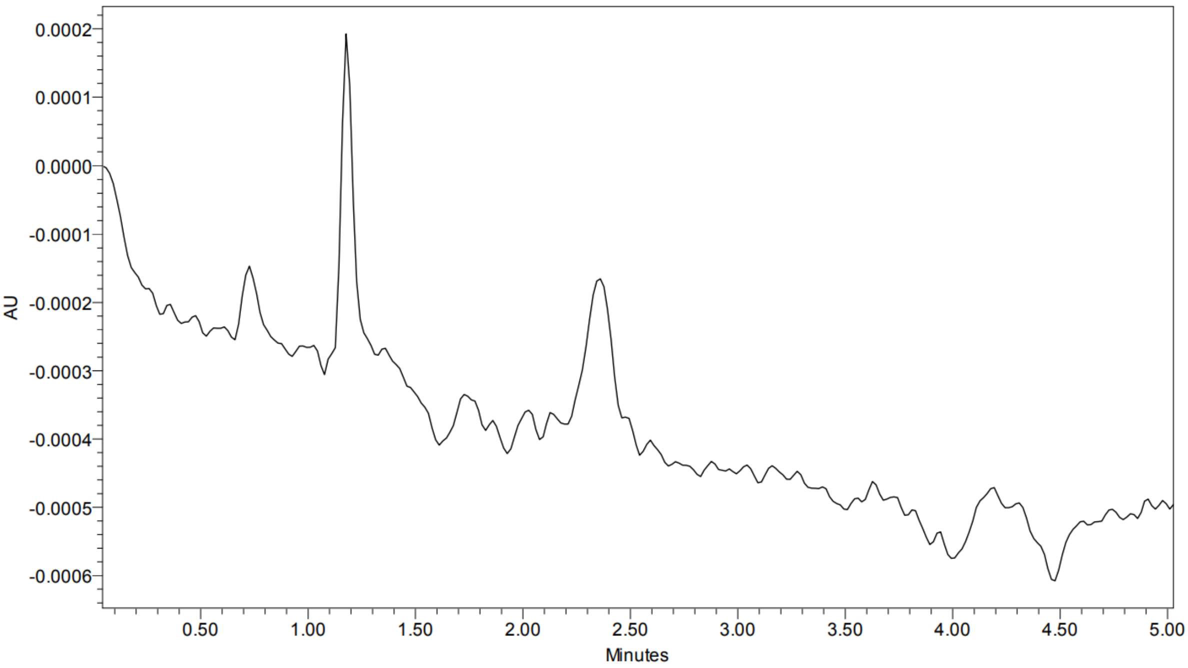

Figure S4: HPLC Chromatogram of Brain blank

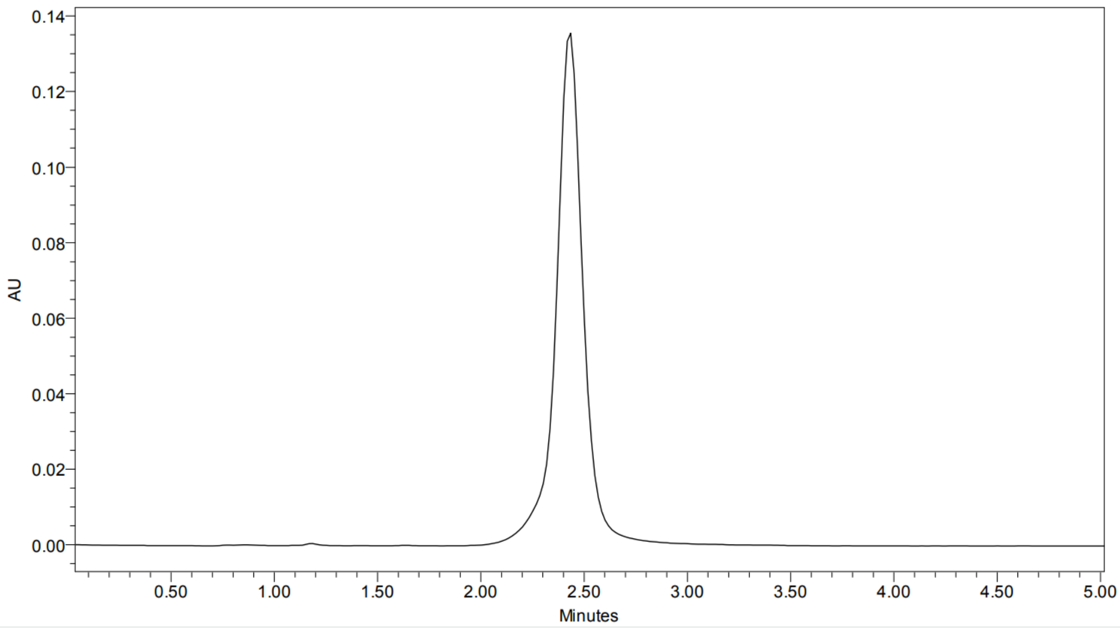

Figure S5: HPLC Chromatogram of Standard piperine in brain

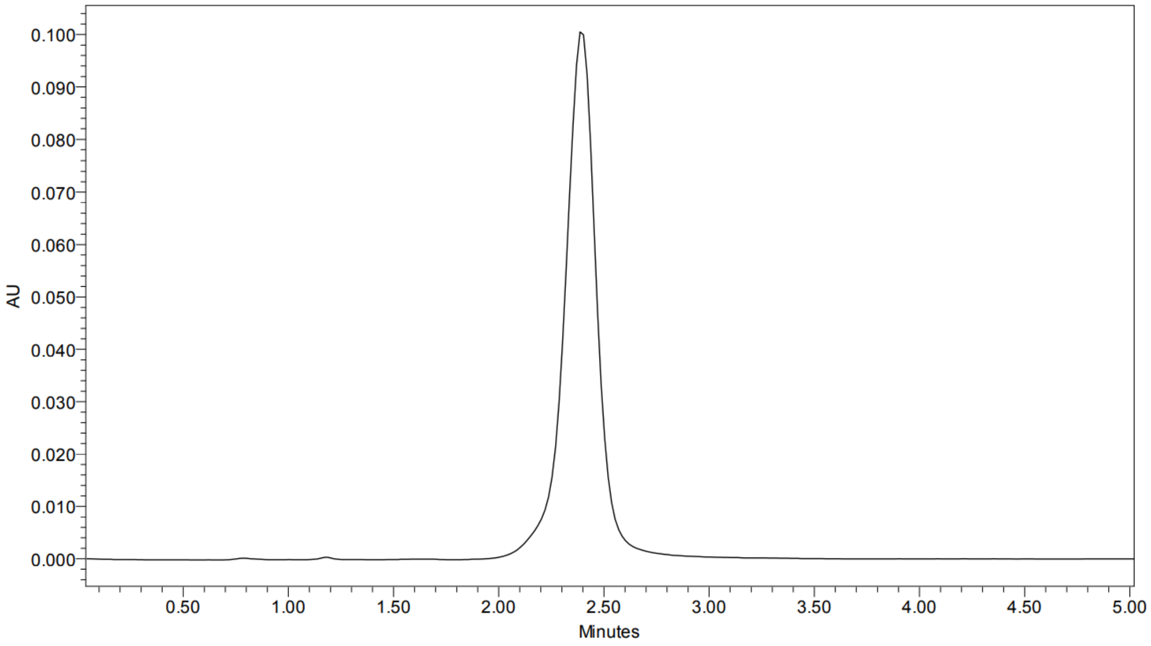

Figure S6: HPLC Chromatogram of PIP-SPL formulation in brain

HPLC graph for Encapsulation efficiency:

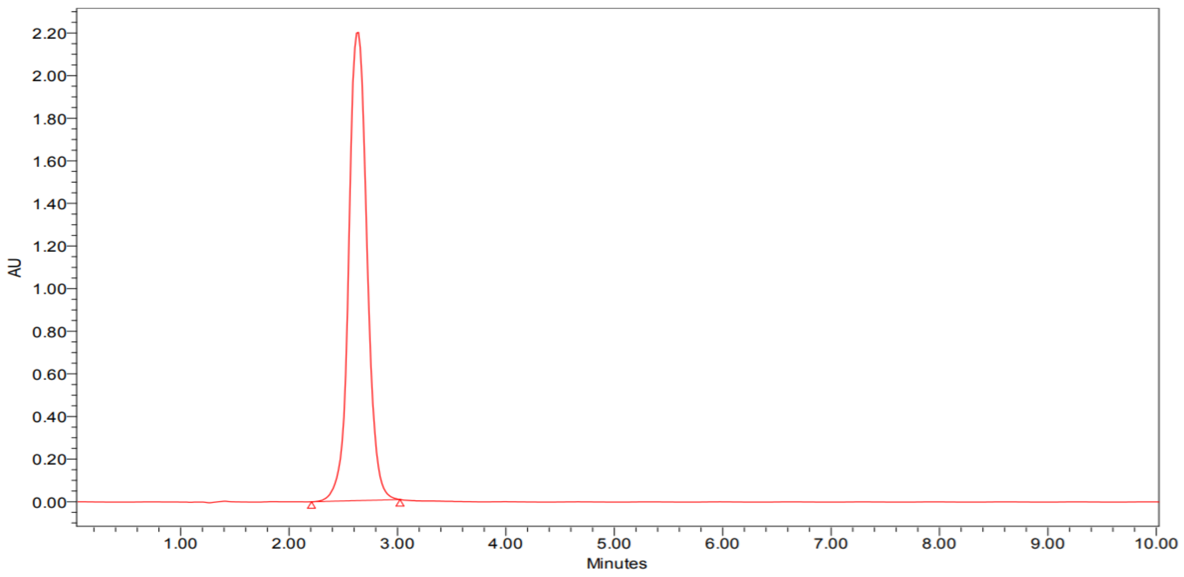

Figure S7: HPLC Chromatogram of standard Piperine

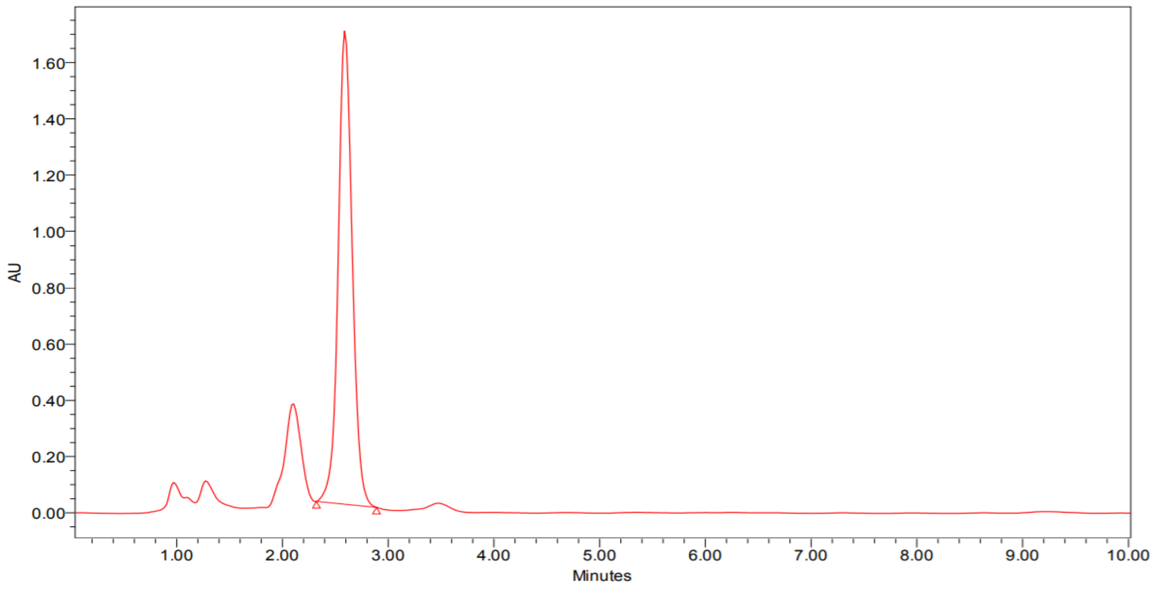

Figure S8: HPLC Chromatogram of PIP-SPLopt formulation
